# Nephrogenic Adenoma of the Urinary Bladder after Kidney Transplantation: Long-Term Follow-Up

**DOI:** 10.1155/2020/8831966

**Published:** 2020-10-16

**Authors:** Iftikhar Khan, Mahmoud Obeid, Nasreen Hasan, Fayyad Jaradat, Bodhisatwa Sengupta, Mahmoud Tabbal, Abdullah Alsaif, Mansour Tawfeeq, Mohammad Al Qahtani

**Affiliations:** ^1^Department of Transplant Surgery, King Fahad Specialist Hospital, Dammam, Saudi Arabia; ^2^Department of Pathology, King Fahad Specialist Hospital, Dammam, Saudi Arabia; ^3^Imam Abdulrahman bin Faisal University, Dammam, Saudi Arabia

## Abstract

Nephrogenic adenoma is a rare lesion that consists of epithelial cells arranged in tubular form, resembling tubules in the renal medulla, and is found usually in the urinary bladder although it can occur anywhere in the transitional epithelium of the lower urinary tract. The first case of nephrogenic adenoma of the urinary bladder was reported before the first kidney transplantation, and the lesion has been reported in patients with and without renal transplantation. The origin of cells in nephrogenic adenoma is debated and has been postulated to arise from cells of embryonic origin or from metaplasia secondary to chronic irritation or from implantation of allograft cells in patients with kidney transplantation. The long-term outcome and potential to convert into malignancy are not established, and therefore, there are no recommendations on how to follow up these patients. We present a case of a patient who was found to have nephrogenic adenoma of the urinary bladder during his second kidney transplantation from a cadaveric donor. He had undergone living donor kidney transplantation previously which subsequently failed. The patient did not manifest any symptoms of nephrogenic adenoma. During a follow-up period of 5 years, he has not manifested any symptoms related to nephrogenic metaplasia. Histopathological examination 5 years after the second transplantation did not show any malignant change. It can be concluded that nephrogenic adenoma is likely to behave in benign fashion post kidney transplantation.

## 1. Introduction

Nephrogenic adenoma (NA) is an uncommon lesion of the lower urinary tract which is found most commonly in the urinary bladder [1]. It presents macroscopically as a polypoid tumor, single or multiple, discovered in patients who usually have hematuria but can be asymptomatic. Microscopic examination reveals a picture similar to renal medullary tubules. There are speculations about the origin of the cells constituting NA as to whether they are metaplastic native cells or implanted cells shed from the kidney in the urine [2, 3]. There are sporadic reports of transformation of NA into malignancy in the nontransplant population. The long-term outcome and malignant transformation potential are not established in the posttransplant population, and therefore, there are no recommendations on follow-up of these patients [4]. We present a case of NA in the urinary bladder of a patient who had undergone kidney transplantation and was followed up for five years. Until we find a specific marker of malignancy for this tumor-like pathology, a case like this may help in formulating a guideline for following up these patients.

## 2. Case Report

**Figure 1:**
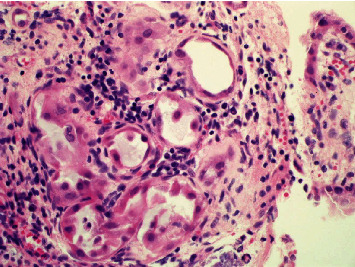
Photomicrograph of the NA in the urinary bladder at the time of second transplantation, showing tubular structures resembling renal medullary tubules.

**Figure 2:**
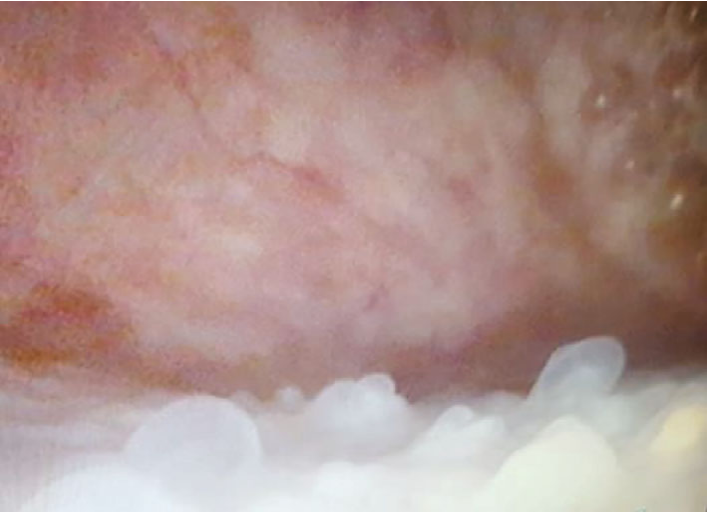
Cystoscopic appearance of NA 5 years after the second transplantation; multiple friable red and white polypoid lesions in the bladder trigone and the roof. Histopathology showed no malignant changes.

A 33-year-old male, on hemodialysis for 4 years, was admitted to our institution for kidney transplantation from a brain-dead 33-year-old male donor. The donor was involved in a road traffic accident. The recipient's primary disease was focal segmental glomerulosclerosis (FSGS), and he also had hypertension. He had undergone living donor kidney transplantation from his brother in the right iliac fossa 11 years ago. His postoperative course after the living donor kidney transplantation was marked by angioplasty for renal artery stenosis and sessions of plasmapheresis for suspected recurrent FSGS. There was no history of hematuria or urinary tract infections. A micturating cystourethrogram (MCUG) showed a grade 2 reflux into the failed allograft. The deceased donor kidney transplantation into the left iliac fossa went uneventfully. The cold ischemia time was 8 hours, and the warm ischemia time was 22 minutes. During the transplantation, the bladder was found to have multiple small polypoid lesions which were biopsied. The histopathology showed features of NA (Figure 1). The patient has been under follow-up for five years with a functional graft. He has no urinary symptoms. Recent cystoscopy revealed mucosal polypoid lesions (Figure 2) which histologically showed features of NA. There were no signs of malignancy.

## 3. Discussion

Since the first report of NA in 1949, physicians have debated the origin of the cells that constitute this peculiar lesion and their potential to transform into malignancy [1–5]. Various theories have been put forward about the origin of the cells including metaplasia secondary to chronic irritation, such as infection and instrumentation, or developmental anomaly in the embryological structures giving rise to the genitourinary tissues. The first case of NA after kidney transplant was reported in 1975 [6]. Mazal et al. demonstrated in renal transplant recipients that the NA cells are actually shed by the allograft by using genetic techniques to show gender match between the NA cells and the donor [3].

Knowing the malignant transformation potential of NA is more relevant to the clinical management of the patients. In the nontransplant setting, there are rare reports of progression of NA into malignancy and researchers consider this transformation an exception and not the rule [4]. There has been no report of malignant transformation in the cases of NA post kidney transplantation. Interestingly, there is a report where the changes of NA reversed with kidney transplantation and restoration of normal bladder function [7].

The duration that NA existed in our patient before the first diagnosis cannot be determined as he was asymptomatic, and no cystoscopy was done. MCUG did not reveal any mucosal abnormality due to the small size of the lesions. The definite follow-up of the patient for five years after the second transplantation demonstrates that the lesion can remain completely asymptomatic for a long term and may behave in a benign fashion in a post kidney transplantation patient. A suggested plan for an asymptomatic patient may be yearly cystoscopy from the first diagnosis for the first five years and then at increasing intervals. Obviously, symptomatic patients will require more frequent evaluations. This suggestion has limitations due to retrospective experience with a single patient.

In conclusion, NA in a post kidney transplant patient is likely to behave as a benign condition. Follow-up may be done by yearly cystoscopy for five years and then with increasing frequency in asymptomatic patients. Further experience is needed to consolidate this recommendation.

## Data Availability

The figures used to support the findings of this study are included within the article.
